# Regulation of Toll-Like Receptor Expression in Human Conjunctival Epithelial Cells

**DOI:** 10.1155/2014/493596

**Published:** 2014-05-25

**Authors:** Jing Li, Melina Setiawan, Hong Wu, Roger W. Beuerman, Peiquan Zhao

**Affiliations:** ^1^Department of Ophthalmology, Xinhua Hospital Affiliated to Shanghai Jiao Tong University School of Medicine, 1665 Kong Jiang Road, Shanghai 200092, China; ^2^Department of Ophthalmology, Second Hospital of Jilin University, Changchun 130041, China; ^3^Singapore Eye Research Institute, 11 Third Hospital Avenue, Singapore 168751

## Abstract

Previous studies showed marked decrease of multiple Toll-like receptor (TLR) expression in corneal and conjunctival epithelial cells upon culture in vitro. The aim of this study was to identify factor(s) which regulate TLR expression. Primary human conjunctival epithelial cells and immortal conjunctival (IOBA-NHC) and corneal epithelial cell lines (HCET) were used. The effect of various cytokines, hypoxia, mechanical wounding, and airlifting culture on TLR expression was examined by quantitative PCR and western blot analysis. Ligand stimulated TLR activation was analyzed. TLR mRNA expression increased modestly when cultured monolayered cells were stimulated by TNF-**α**, IL-1**α**, IL-1**β**, IL-6, IL-8, IFN-**γ** (about 2-fold), hypoxia (2.1- to 4.8-fold selectively), and wounding (3.1- to 9.3-fold). In airlifted multilayered cells, TLR expression increased 7.8- to 25.9-fold compared to monolayered cells. Airlifted cells showed increased response to low concentrations of lipopolysaccharide (LPS) and peptidoglycan (PGN) stimulation. NF**κ**B inhibition prevented the formation of cell sheets and led to the collapse of already-formed multilayered structure and the simultaneous reduction of TLR mRNA level. In conclusion, our study showed that the conjunctival epithelial cell expressed TLR was sensitive to various stimulants, and a multilayered epithelium-like structure was needed to maintain TLR expression.

## 1. Introduction

Toll-like receptors (TLRs) are a family of pattern-recognition receptors [1]. Ten TLR proteins have been identified in human cells. Each TLR binds specific pathogen-associated molecular patterns (PAMPs) of viruses, bacteria, fungi, and parasites. Some TLRs, such as TLR3 and TLR4, also recognize damage-associated molecular patterns (DAMPs) released by injured cells [2]. The activation of TLR and its associated signaling pathway leads to a broad range of inflammatory responses mediated mainly by increased secretion of cytokines [3].

Ocular surface epithelial cells, namely, conjunctival and corneal epithelial cells, were known to express multiple TLRs [4–6]. Cultured monolayer cells were most often used to study the expression, function, and regulation of TLR in epithelial cells. While the most TLR expressed in epithelial cells showed responses to its ligands in cultured cells, contradictory results were reported on the biological activity of TLR2 and TLR4 in corneal and conjunctival epithelial cells [7–9]. In a previous study, we quantified and compared the expressions of multiple TLRs in primary cultured human conjunctival epithelial cells and conjunctival epithelium tissue and found that TLR expressions in cultured cells were much lower than those in undigested tissue [6]. Here, we explored the effect of multiple factors on TLR expression and report that the multilayered structure was a key factor to maintain a normal level of TLR expression in conjunctival and corneal epithelial cells.

## 2. Materials and Methods

### 2.1. Reagents

Unless otherwise specified, all cell culture medium and supplements were purchased from Life Technologies (Carlsbad, CA, USA). All plasticware for cell culture was purchased from Greiner Bio-One (Frickenhausen, Germany). General reagents for sodium dodecyl sulfate polyacrylamide gel electrophoresis (SDS-PAGE) and western blot were purchased from Bio-Rad (Hercules, CA, USA). Lipopolysaccharide (LPS) isolated from* Pseudomonas aeruginosa* and peptidoglycan (PGN) isolated from* Bacillus subtilis* were purchased from Sigma-Aldrich (St. Louis, MO, USA). 5-Aminosalicylic acid (5-ASA, NF*κ*B inhibitor) was purchased from Santa Cruz Biotechnology (Dallas, TX, USA).

### 2.2. Primary Conjunctival Epithelial Cell Isolation and Culture

Primary human conjunctival epithelial cells were isolated from cadaver conjunctival tissue obtained from Singapore Eye Bank as described before [10]. Briefly, after antibiotics/PBS washing, the conjunctival tissue was cut into small pieces and placed on cell culture plate with one drop of full medium which contained equal volume of DMEM and F12, 10% fetal bovine serum (FBS), 0.5 *μ*g/mL hydrocortisone, 10 nM cholera toxin, 10 ng/mL human epidermal growth factor (hEGF), 5 *μ*g/mL insulin, and antibiotics. Epithelial cell outgrowth was observed 2-3 days later, and the culture was maintained for 4-5 days before the tissues were discarded. The cells were then submerged-cultured in the same medium for further propagation. Passage 2 to 3 cells were used in this study. The study protocol was approved by the Institutional Review Board of Singapore Eye Research Institute and followed the tenets of the Declaration of Helsinki.

### 2.3. Conjunctival and Corneal Epithelial Cell Lines

Immortalized human conjunctival epithelial cell (IOBA-NHC) was a gift from Dr. Diebold at the University of Valladolid, Spain [11]. SV40 large T-antigen immortalized human corneal epithelial cell (HCET) was purchased from Riken Cell Bank (Ibaraki, Japan) [12]. Both cells were cultured in the same medium as the primary conjunctival epithelial cells.

### 2.4. Cell Treatment

#### 2.4.1. Culture Supplement

A basic medium was prepared first which contained equal volume of DMEM and F12 with antibiotics. The following supplements were added individually to the basic medium: 1 *μ*g/mL bovine insulin, 2 ng/mL recombinant hEGF, 0.5 *μ*g/mL hydrocortisone, 10% FBS, and 0.1 *μ*g/mL cholera toxin. The isolated primary human conjunctival epithelial cells were submerged in the above medium for 24 hrs, and TLR mRNA expression was compared to cells grown in full and basic medium.

#### 2.4.2. Airlifted Culture

Airlifted culture refers to the culture condition in which cells were grown at the air-medium interphase. To achieve this, submerged-cultured cells were trypsinized and seeded on BioCoat collagen I coated 6-well inserts with 3.0 micron pore (BD Biosciences, San Jose, CA, USA). Cells in the inserts were submerged in the full medium for the first 24 hrs and continued in a medium which contained DMEM : F12 (3 : 1, V : V) with the same supplement as in the full medium, and the volume of medium was reduced so that only the bottom of the insert was in contact with the medium (about 1 mL per well). Epithelial cells started to form multilayered sheet after 3 days. The culture was maintained for 7–12 days depending on the progression of cell stratification, and the culture medium was changed every day.

#### 2.4.3. Cytokine Stimulation

Selected cytokines were added to 80–90% confluent submerged cells for 24 hrs before the supernatant was harvested for cytokine secretion analysis.

#### 2.4.4. Cell Wounding

Eight to ten parallel scratches were made to 80–90% confluent submerged cells at about 1 cm space using a sterile surgical blade. Cells were then washed with PBS and given fresh medium after wounding. Supernatant was harvested 24 hrs later for cytokine secretion analysis.

#### 2.4.5. Hypoxia Stimulation

Hypoxia stimulation was achieved by culturing submerged cells in 37°C incubator with 95% nitrogen and 5% carbon dioxide for 24 hrs.

### 2.5. Quantitative PCR (qPCR) Analysis

Total RNA was extracted using NucleoSpin RNA II (Macherey-Nagel, Germany) and transcribed into cDNA using SuperScript III Reverse Transcriptase from Life Technologies. qPCR was performed using Taqman real-time PCR reagents as previously described [6]. *β*-Actin was used as internal control. The mRNA expression was calculated by the comparative expression methods of 2^(−*δδ*Ct)^, where *δ*Ct is the threshold cycle and *δδ*Ct = *δ*Ct_airlifted_ − *δ*Ct_submerged_.

### 2.6. Western Blot Analysis

Cells were lysed in RIPA buffer containing 10 mM Tris pH 7.5, 150 mM NaCl, 1% sodium deoxycholate, 1% Triton X-100, 1 mM EDTA, and a protease inhibitor cocktail (Roche Diagnostics Asia Pacific, Singapore). Protein concentration was measured by Pierce Micro BCA Protein Assay Kit (Thermo Scientific, Rockford, IL, USA). Thirty micrograms of the total lysates was resolved on SDS-PAGE and transferred to nitrocellulose membrane. Blots were probed with the following antibodies at 4°C overnight: goat anti-TLR1 antibody (R&D Systems, Minneapolis, MN) at the concentration of 2 ng/lane; monoclonal anti-TLR2 and anti-TLR3 at the dilution of 1 : 100 (Imgenex, San Diego, CA); rabbit anti-TLR4, rabbit anti-TLR5, and goat anti-TLR6 antibodies (Santa Cruz Biotechnology, Santa Cruz, CA) at the dilution of 1 : 200; and monoclonal anti-TLR9 antibody (Abcam, Cambridge, UK) at the concentration of 1 *μ*g/mL. Horseradish peroxidase conjugated species-specific secondary antibody (Santa Cruz Biotechnology, Santa Cruz, CA) incubation was carried out at room temperature for 1 hr (1 : 2000 for anti-rabbit antibody sc-2030; 1 : 2000 for anti-mouse antibody sc-2005; and 1 : 5000 for anti-goat antibody sc-2350). The resulting immune complex was visualized using SuperSignal chemiluminescent substrates (Thermo Scientific, Rockford, IL, USA) and exposed to X-ray film.

### 2.7. IL-6 and IL-8 Secretion Analysis

IL-6 and IL-8 secretion was analyzed in the culture medium using OptEIA (BD Biosciences, USA) and corrected by total cellular protein and total medium volume. For each experiment, an aliquot of the culture medium was saved, briefly spun, and stored at −80°C for ELISA analysis. Total medium volume (mL) at the time of harvesting was recorded. Cells were washed in PBS twice and lysed, and total protein content was measured with Pierce Micro BCA Protein Assay Kit. IL-6 and IL-8 secretion was expressed as follows: cytokine concentration (pg/mL) multiplied by total medium (mL) volume divided by total cell protein content (mg). The final unit was pg cytokine/mg cell protein.

### 2.8. NF*κ*B Activity Analysis

The activities of p65 and p50 subunits of NF*κ*B were measured by ELISA analysis using 96-well plates precoated with NF*κ*B-binding DNA consensus sequence (Pierce, Rockford, IL). Only the active form of p65 and p50 binds to the immobilized DNA sequence, and the bound protein was subsequently detected by specific primary antibody against p65 and p50 followed by HRP conjugated secondary antibody. The chemiluminescence signal was measured by Victor X3 microplate reader (Perkin Elmer, China). Data were expressed as the mean ± SE.

### 2.9. Statistical Analysis

Data were expressed as mean ± standard deviation and analyzed by analysis of variance (ANOVA) after Levene's test for homogeneity, followed by the Fisher least significant difference (LSD) test. A probability level of *P* < 0.05 was considered as statistically significant.

## 3. Results

### 3.1. Culture Medium Supplements Had No Effect on TLR Gene Expression in Primary Cultured Conjunctival Epithelial Cells

No changes in TLR mRNA expression were measured by qPCR in monolayered cells cultured in the basic medium with additional insulin, recombinant hEGF, hydrocortisone, FBS, or cholera toxin (data not shown).

### 3.2. Airlifting Culture Stimulated TLR mRNA and Protein Expression

**Figure 1:**
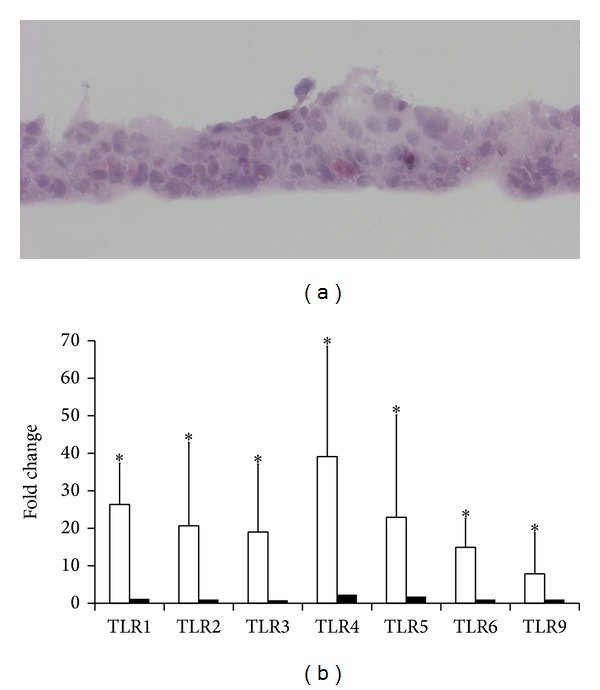
TLR mRNA expression in airlifted primary human conjunctival epithelial cells. (a) Hematoxylin and eosin staining of a representative cell sheet 7 days after airlifting culture. Typically, the cell sheet was composed of 3-4 layers of cells. Cells at the bottom were mostly cuboidal and more compact than the cells at the upper layers. (b) Averaged fold increase of individual TLR mRNA in airlifted (white bars) and replated conjunctival epithelial cells (black bars). Cells were harvested 10 days after airlifting culture. Half of the cells were replated to 6-well plate and submerged-cultured for 24 hrs in 1 : 1 medium with full supplement before being lysed for RNA extraction. TLR mRNA level was determined by qPCR and compared to the same batch of cells submerged-cultured. Three different batches of cells were analyzed, and the fold increase was averaged. *Y* error bars represent the standard deviation of the averaged results. Asterisks (∗) denote significant difference (*P* < 0.05) between airlifted and replated cells.

Primary conjunctival epithelial cells formed multilayered, stratified structure when airlifted (Figure 1(a)). Increased TLR mRNA expression was first detected by qPCR in cells 3 days after airlifting and continued to increase as cells form stratified sheets.  Table 1 lists the *δ*Ct values for TLR mRNA in submerged and 10-day airlifted primary human conjunctival cells. The averaged fold increase of each TLR mRNA is presented in Figure 1(b). TLR4 mRNA expression as detected in only one batch of submerged primary cells but in all airlifted cells.

Next, we replated the airlifted primary cells on 6-well plate and maintained at submerged condition. Less than 10% of the cells adhered to the culture dish after 24 hrs. TLR mRNA expression decreased to levels prior to airlifting culture (Figure 1(b)). In cells which had no detectable amount of TLR4 mRNA, it remained positive when the airlifted cells were replated.

**Figure 2:**
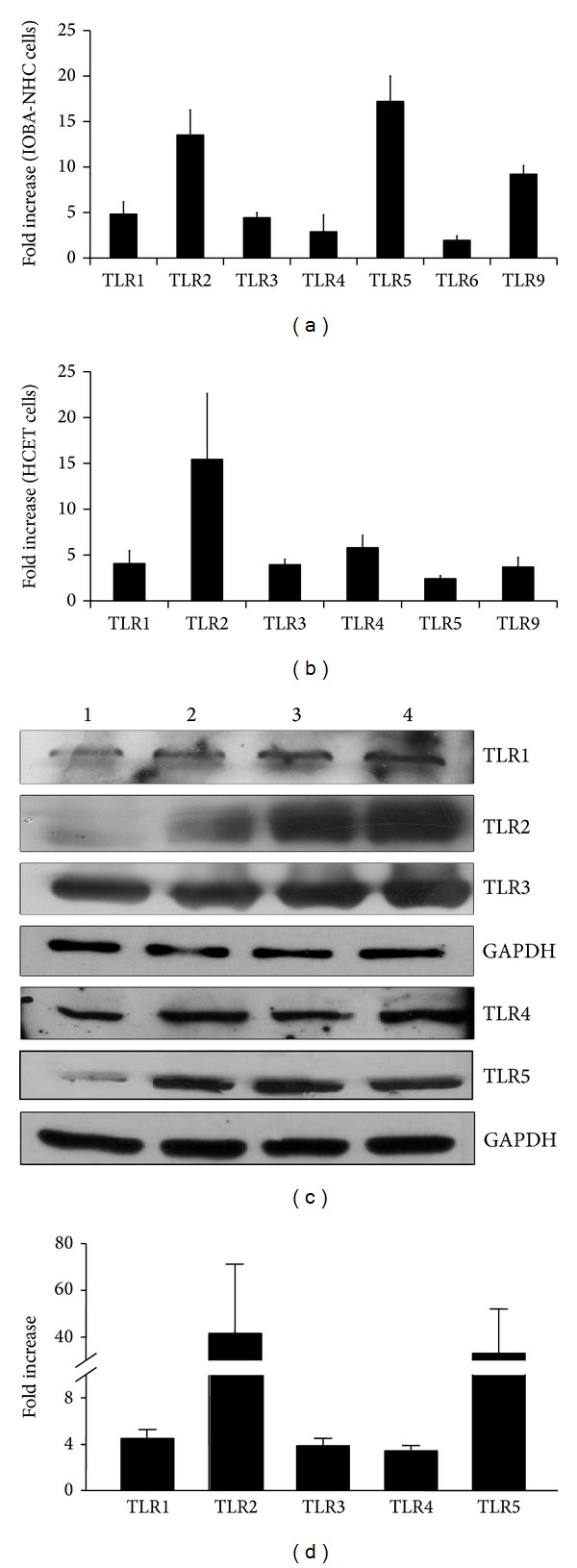
TLR mRNA and protein expression in airlifted immortal human corneal (HCET) and conjunctival epithelial cells (IOBA-NHC). (a) Averaged fold increase of TLR mRNA in IOBA-NHC cells. The increase was statistically significant for each TLR. (b) Averaged fold increase of TLR mRNA in HCET cells. The increase was statistically significant for each TLR. Cells were airlifted for 10 days. TLR mRNA level was determined by qPCR and compared to submerged cells. The experiment was repeated 3 times. *Y* error bars represent the standard deviation of the averaged results. (c) Micrographs of a representative western blot showing TLR protein expression in IOBA-NHC cells. Lane 1: submerged-cultured; lane 2: 3 days after airlifting culture; lane 3: 7 days after airlifting culture; lane 4: 10 days after airlifting culture. Glyceraldehyde 3-phosphate dehydrogenase (GAPDH) protein was probed as loading control. (d) Averaged TLR protein increase in IOBA-NHC cells 10 days after airlifting culture compared to submerged-cultured cells. X-ray films from 2 independent western blot experiments were scanned in a densitometer, and the results were averaged. The increase was statistically significant for each TLR protein tested.

The same experiments were repeated in immortalized human corneal (HCET) and conjunctival epithelial cells (IOBA-NHC). Differences in TLR mRNA expression were noticed (Table 1). TLR6 mRNA expression was not detected in HCET cells by qPCR. Airlifting culture induced further increase of all TLR mRNA in both cell lines, however, at a smaller magnitude than the primary cells (Figures 2(a) and 2(b)).

Due to the limited availability of primary human cells, we used IOBA-NHC cells to compare the changes of TLR protein expression before and after airlifting culture. Western blot analysis revealed increased TLR1, TLR2, TLR3, TLR4, and TLR5 expression, which largely matched the increase of the respective mRNA (Figures 2(c) and 2(d)). Because TLR2 and TLR5 were barely detectable in submerged-cultured IOBA-NHC cells, the calculated increase for the protein was greater than that for its respective mRNA. Similar results were obtained from HCE cells (data not shown).

### 3.3. Airlifted Conjunctival Epithelial Cells Responded to Low Concentrations of LPS and PGN Stimulation

To test the effect of increased TLR expression in airlifted cells, we compared LPS and PGN stimulated IL-6 and IL-8 secretion in primary conjunctival epithelial cells cultured under submerged and airlifted conditions.

**Figure 3:**
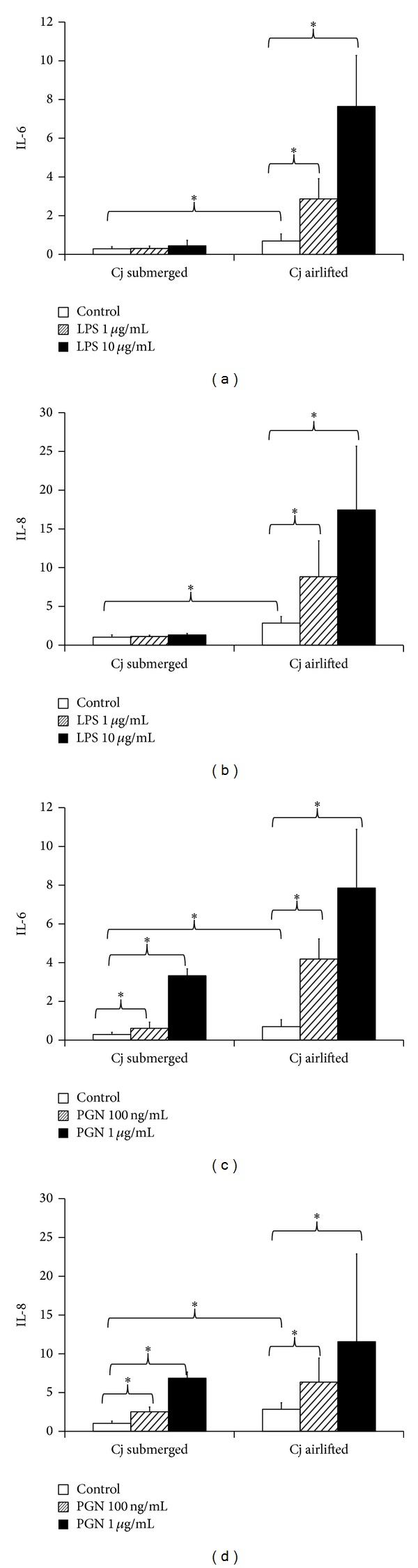
LPS and PGN stimulated IL-6 and IL-8 secretion in submerged and airlifted primary conjunctival epithelial cells. (a) LPS stimulated IL-6 secretion. (b) LPS stimulated IL-8 secretion. (c) PGN stimulated IL-6 secretion. (d) PGN stimulated IL-8 secretion. IL-6 and IL-8 concentrations were expressed as pg/mL medium/mg total cellular protein. Asterisks (∗) denote significant difference (*P* < 0.05 by paired *t*-test) between two groups. *Y* error bars represent the standard deviation of the averaged results from 3 different experiments.

Compared to submerged culture, airlifting required less medium level and had higher cell density. To correct for these differences, we calculated cytokine concentrations in the unit of pg/mL medium/mg total cell protein. We found that both IL-6 and IL-8 concentrations were significantly higher in the airlifting culture medium than in the submerging culture medium without added ligands (Figure 3).

When incubated with 1 and 10 *μ*g/mL LPS for 24 hrs, only airlifted cells showed increased IL-6 and IL-8 secretion. No significant changes were measured in the submerged-cultured cells (Figures 3(a) and 3(b)).

When incubated with 100 ng/mL and 1 *μ*g/mL PGN for 24 hrs, the airlifted cells showed more increase of both IL-6 and IL-8 compared to the submerged cells (Figures 3(c) and 3(d)).

### 3.4. Airlifted Conjunctival Epithelial Cells Were Sensitive to NF*κ*B Inhibition

In an attempt to explore the mechanisms underlying increased TLR expression in airlifted epithelial cells, we studied the response of submerged and airlifted primary cultured conjunctival epithelial cells and IOBA-NHC cells to the treatment of 5-ASA, an inhibitor of NF*κ*B.

**Figure 4:**
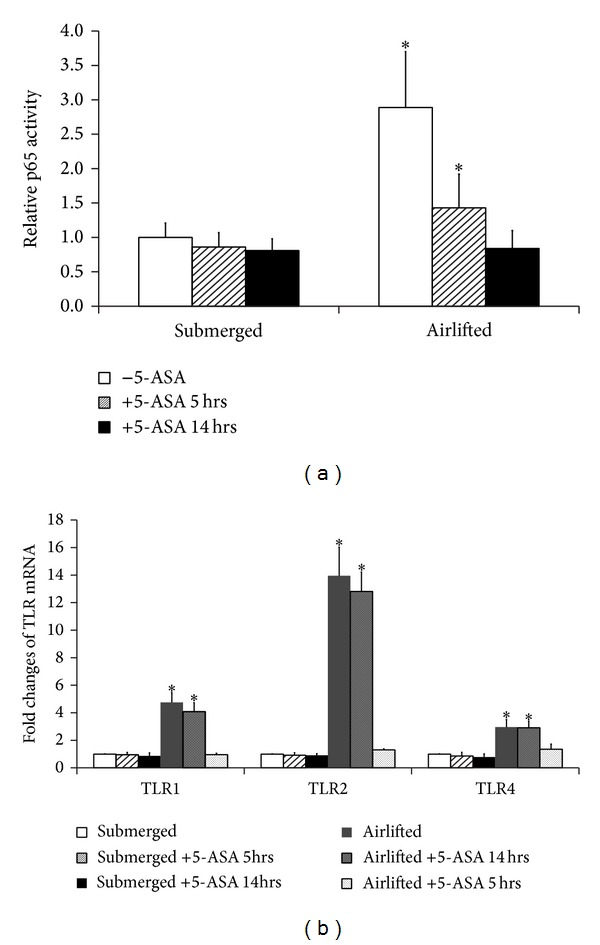
The effect of NF*κ*B inhibition on airlifted IOBA-NHC TLR expression. (a) Relative activity of p65 in submerged and airlifted IOBA-NHC cells with and without 5-ASA. p65 activity in submerged monolayer cells without 5-ASA was set as 1, and the activities of other cell conditions were compared to it. Asterisks (∗) denote significant difference (*P* < 0.05) between airlifted and submerged cells under the same treatment. (b) Fold changes of selective TLR mRNA expression by qPCR. Individual TLR expression in submerged monolayer cells without 5-ASA was used as control to calculate the fold change. Asterisks (∗) denote significant difference (*P* < 0.05) between airlifted and submerged cells under the same treatment. All experiments were repeated three times, and the data were averaged. *Y* error bars represent the standard deviation of the averaged results.

Neither primary cells nor immortal cells were able to form multilayered sheets in the presence of 5-ASA. When 5-ASA was added to the airlifted multilayered cells (4-5 days after airlifting culture), we observed an almost complete collapse of the multilayered structure 14 hrs after the drug treatment. Airlifted IOBA-NHC cells showed higher NF*κ*B activity than submerged cells (Figure 4(a)). At 5 hrs after 5-ASA treatment, IOBA-NHC cells showed decreased NF*κ*B activity but little changes in TLR expression. TLR mRNA was reduced to close to the pre-airlifting level at 14 hrs after the treatment (Figure 4(b)). However, we found no significant changes of TLR mRNA with 5-ASA treatment in monolayered cells.

### 3.5. Proinflammatory Cytokines Stimulated TLR mRNA Expression in Conjunctival Epithelial Cells

**Figure 5:**
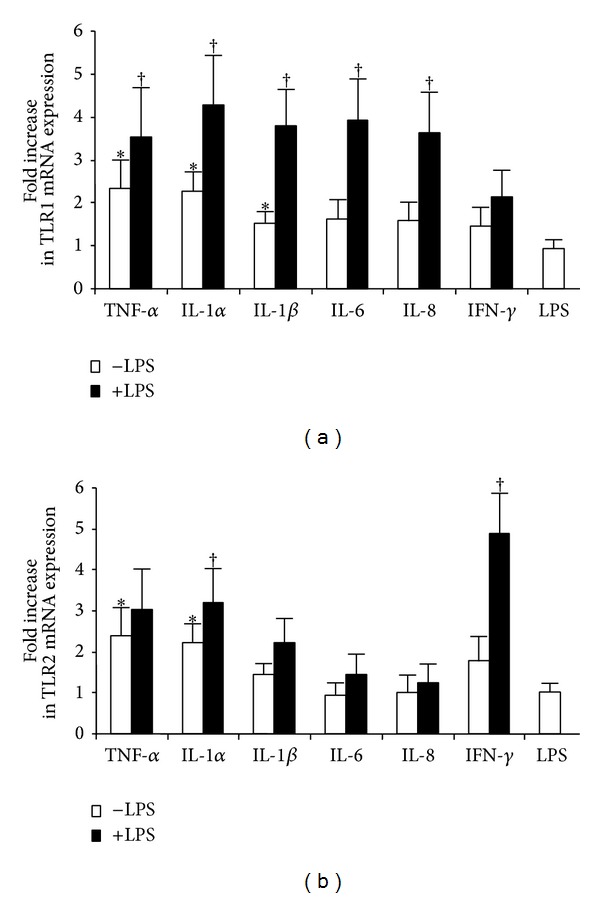
TNF-*α* (10 ng/mL), IL-1*α* (10 ng/mL), IL-1*β* (10 ng/mL), IL-6 (10 ng/mL), IL-8 (10 ng/mL), and IFN-*γ* (40 ng/mL) stimulated TLR1 and TLR2 mRNA expression in submerged primary conjunctival epithelial cells with and without 50 ng/mL LPS. TLR expression in cells cultured in normal medium was used as reference for the calculation of fold increase. Open bars represent the averaged increase when cells were stimulated with cytokine alone. Solid black bars represent the averaged increase when cells were costimulated with indicated cytokines and LPS. The experiments were repeated in three different batches of cells, and the averaged results were presented. *Y* error bars represent the standard deviation of the averaged results. Asterisks (∗) denote significant difference when cytokine alone was compared to that without stimulation, and (†) denotes significant difference when cytokine alone was compared to that with LPS.

We examined TLR mRNA expression in submerged primary cultured conjunctival epithelial cells in the presence of 10 ng/mL TNF-*α*, IL-1*α*, IL-1*β*, IL-6, and IL-8; 40 ng/mL IFN-*γ* alone; and 50 ng/mL of LPS. Different batches of primary cells showed different responses to cytokine stimulation. Overall, no consistent or significant changes of TLR4 and TLR6 mRNA (larger than 2-fold increase in all cells tested) were detected when cells were stimulated with the above cytokines. Small increase (2-3-fold) of TLR1, TLR2, TLR3, TLR5, and TLR9 mRNA was observed when stimulated with TNF-*α* and IL-1*α*, but not with IL-1*β*, IL-6, or IL-8. On average, IFN-*γ* stimulated the expression of TLR3 and TLR9 mRNA by more than 2-fold, but not other TLRs. While 50 ng/mL LPS alone did not have significant effect on TLR mRNA expression, it showed synergistic effect when costimulated with some cytokines. The changes of TLR1 and TLR2 mRNA were shown as representatives (Figure 5).

### 3.6. Hypoxia Stimulated TLR mRNA Expression in Conjunctival Epithelial Cells

**Figure 6:**
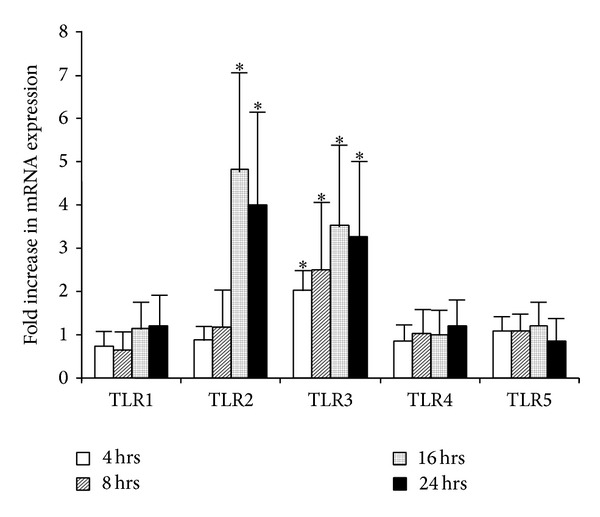
Hypoxia stimulated TLR mRNA expression in submerged primary conjunctival epithelial cells. Cells were cultured in 95% N_2_ and 5% CO_2_ at 37°C for indicated duration before being harvested for RNA extraction and qPCR. The experiment was repeated in 3 different batches of primary cells, and the averaged changes were shown. *Y* error bars represent the standard deviation of the averaged results. Asterisks (∗) denote significant difference when compared to cells under normoxia condition.

Hypoxic culture stimulated TLR2 and TLR3 mRNA expression in submerged-cultured primary human conjunctival epithelial cells (Figure 6). A 4.81-fold increase of TLR2 mRNA was observed in cells 16 hrs after hypoxia culture and remained increased by 4.00-fold at 24 hrs (Figure 6). TLR3 mRNA increased 2.04-fold at 4 hrs after hypoxia, peaked at 16 hrs, and remained elevated at 24 hrs. No changes of TLR1, TLR4, and TLR5 mRNA expression were observed.

### 3.7. Wounding Stimulated TLR mRNA Expression in Conjunctival Epithelial Cells

**Figure 7:**
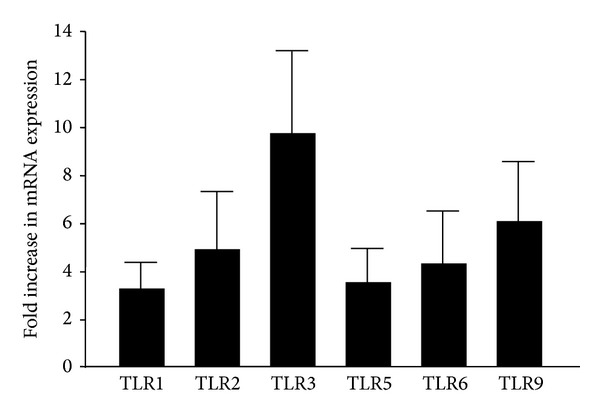
Wounding stimulated TLR expression in submerged primary conjunctival epithelial cells. Confluent cells were scratched by sterile surgical blade at about 1 cm interval and harvested 24 hrs later for RNA extraction and qPCR analysis. The experiment was repeated in 3 different batches of primary cells, and the averaged changes were shown. *Y* error bars represent the standard deviation of the averaged results. The increase was statistically significant for each TLR.

Wounding stimulated the expression of most TLR mRNA in submerged-cultured cells (Figure 7). Specifically, TLR3 mRNA increased 9.3-fold; TLR9, TLR2, and TLR6 increased 6.2-, 4.8-, and 4.2-fold, respectively. TLR5 and TLR2 mRNA increased 3.3- and 3.1-fold. However, TLR4 mRNA remained unexpressed in all three batches of primary cells tested.

## 4. Discussion

In this study, we showed that TLR expression in cultured human conjunctival epithelial cells could be stimulated by proinflammatory cytokines, hypoxia, wounding, and airlifting culture, but not by culture medium supplement. Among these, airlifting culture had the most powerful effect on TLR expression. In airlifted conjunctival cells, the expression of TLR mRNA and protein was similar to what we have previously reported in vivo. Increased TLR expression in airlifted cells led to enhanced cytokine responses to low concentrations of TLR ligands, including LPS. Our study also showed that both the formation of multilayered cell sheet and increased TLR expression were sensitive to the inhibition of NF*κ*B.

Our data suggested that TLR expression was intrinsically associated with multilayered structure formed under airlifting culture, which required NF*κ*B activation. Airlifting culture of conjunctival and limbal epithelial cells is an effective and commonly used approach to achieve a multilayered epithelium-like structure in vitro. The multilayered epithelial cell sheets resulting from airlifting culture have been used as tissue graft on various ocular surface engineering applications, mostly using amniotic membrane as support [13, 14]. Significant changes occur when epithelial tissue was digested and cells were cultured in monolayer in vitro, especially the loss of cell-cell connection. Our results indicated that the formation of multilayered structure was needed for the proper expression of TLR in conjunctival epithelial cells. Activation of NF*κ*B was required for the formation of multilayered cell sheets. Due to the sensitive nature of airlifting culture, we were not able to further dissect the association between the formation of cell-cell connection and TLR expression at this time. However, this is not the first study which showed association between TLR expression and intact epithelial cell structure. Previous studies reported that the intestinal epithelium of TLR2 and MyD88 knockout mouse was prone to stress-induced tight junction disruption, suggesting a relationship between functional TLR and the integrity of the epithelium [15, 16]. Low levels of TLR expression were also reported in cultured intestinal and lung epithelial cells. Unlike ocular surface, intestinal epithelium and lung epithelium contain polarized single layer epithelial cells. It would be interesting to investigate the changes of TLR in these cells under similar culture conditions.

The stimulation by proinflammatory cytokines, hypoxia, and mechanical wounding mimics the stress to which ocular surface epithelial cells may be exposed in vivo. The changes of TLR mRNA expression in cultured primary conjunctival cells demonstrated in this study suggested that epithelial cell-expressed TLR was responsive to hypoxia and wounding. The effect of proinflammatory cytokines on individual TLR mRNA expression was relatively small. We also found several studies which supported our results. For example, two recent studies described increased multiple TLR expression in mouse corneal and conjunctival epithelial cells of dry eye disease models [17, 18]. Proinflammatory cytokines also showed stimulatory effect on human airway epithelial cell TLR2 expression [19]. However, the effect of hypoxia on individual TLR expression seemed to be different; that is, the expression of TLR2 and TLR3 was increased, while TLR1, TLR4, and TLR5 were not changed. Contradictory results were also reported on the effect of hypoxia on TLR4 expression in macrophage, endothelial cells, and corneal epithelial cells [20–22]. Further studies were needed to clarify the mechanism which mediates the effect of hypoxia on TLR expression.

In conclusion, this study identified multiple factors which regulated TLR expression in ocular conjunctival epithelial cells. Most importantly, our data suggested the existence of a coordinated reinforcement of both physical and molecular defense mechanisms during the formation of epithelial structure. Therefore, we propose that multilayered cell sheet, instead of submerged-cultured monolayer cells, is a better system to evaluate the biological activities of epithelial cell-expressed TLR.

## Figures and Tables

**Table 1:** ΔCt of individual TLR mRNA in submerged and airlifted primary human conjunctival epithelial cells, immortalized human conjunctival epithelial cells (IOBA-NHC), and immortalized human corneal epithelial cells (HCET).

mRNA	Primary conjunctival epithelial cells (*n* = 3)		IOBA-NHC cells		HCET cells	
	Submerged	Airlifted	Submerged	Airlifted	Submerged	Airlifted
TLR1	10.97 ± 1.08	6.27 ± 0.72	7.02 ± 0.36	4.77 ± 0.42	10.58 ± 0.03	8.55 ± 0.14
TLR2	10.51 ± 1.84	6.14 ± 1.32	8.92 ± 0.24	5.17 ± 0.56	5.93 ± 0.38	1.98 ± 0.18
TLR3	9.13 ± 1.93	4.88 ± 1.23	4.69 ± 0.18	2.57 ± 0.38	4.63 ± 0.17	2.65 ± 0.26
TLR4	14.93 (*n* = 1)	9.74 ± 1.01	3.06 ± 0.14	1.57 ± 0.30	7.25 ± 0.04	4.71 ± 0.10
TLR5	10.61 ± 2.08	6.28 ± 1.13	10.85 ± 1.79	6.75 ± 0.18	5.64 ± 0.17	4.36 ± 0.09
TLR6	12.41 ± 1.02	8.51 ± 0.53	5.39 ± 0.48	4.48 ± 0.51	ND	ND
TLR9	13.32 ± 1.19	10.36 ± 0.78	9.54 ± 0.73	6.35 ± 0.16	10.10 ± 0.46	8.21 ± 0.79

ΔCt was calculated using *β*-actin as internal control. Data was presented as average ± standard deviation. The experiment was performed three times for each condition. For primary cells, the results shown were the averaged *δ*Ct of three different batches of cells. “Submerged” refers to submerged-cultured cells at 80–90% confluence. “Airlifted” refers to airlifted culture at 10 days. ND stands for nondetected.
